# Role of Quantitative CEUS in the Diagnosis of Peripheral Pulmonary Lesions: A Systematic Review

**DOI:** 10.3390/cancers17101697

**Published:** 2025-05-18

**Authors:** Andrea Boccatonda, Alice Brighenti, Sofia Maria Bakken, Damiano D’Ardes, Cosima Schiavone, Fabio Piscaglia, Carla Serra

**Affiliations:** 1 Diagnostic and Therapeutic Interventional Ultrasound Unit, IRCCS Azienda Ospedaliero-Universitaria di Bologna, 40138 Bologna, Italy; alice.brighenti3@studio.unibo.it (A.B.); sofiabakkenborel@gmail.com (S.M.B.); carla.serra@aosp.bo.it (C.S.); 2 Department of Medicine and Aging Science, Institute of “Clinica Medica”, “G. D’Annunzio” University of Chieti-Pescara, 66100 Chieti, Italy; damiano.dardes@unich.it; 3 Department of Medicine and Aging Science, “G. D’Annunzio” University of Chieti-Pescara, 66100 Chieti, Italy; cosima.schiavone@gmail.com; 4 Department of Medical and Surgical Sciences (DIMEC), University of Bologna, 40138 Bologna, Italy; fabio.piscaglia@unibo.it; 5 Division of Internal Medicine, Hepatobiliary and Immunoallergic Diseases, IRCCS Azienda Ospedaliero-Universitaria di Bologna, 40138 Bologna, Italy

**Keywords:** lung, CEUS, cancer, metastasis, lesion

## Abstract

**Background/Objectives:** This systematic review describes a largely descriptive synthesis of studies investigating the diagnostic performance of quantitative contrast-enhanced ultrasound (CEUS) in differentiating benign from malignant peripheral pulmonary lesions. **Methods**: Formal quantitative pooling of effect sizes was not feasible due to variability in outcome measurements and reporting. **Results:** Combining CEUS parameters with real-time on-site evaluation (ROSE) substantially improved percutaneous biopsy success rates. In one comparative study, biopsy yield reached 97.62% with CEUS, versus 84% using conventional ultrasound, while complications remained minimal. Other investigations focused on the discriminatory value of specific time-based indices (e.g., AT ≥ 10 s, lesion-lung AT difference ≥ 2.5 s) or complex multi-parameter models. A notable large study demonstrated that a six-parameter logistic regression model achieved near-excellent discrimination, with C-statistics exceeding 0.97 for both development and validation cohorts, outperforming single-threshold approaches. Nevertheless, certain findings emphasize that no single indicator—particularly arrival time alone—reliably distinguishes benign from malignant lesions, given the diverse vascular patterns and histological subtypes involved. TDOA-based analyses proved more promising, as malignant lesions generally exhibit a delayed but robust bronchial arterial supply and rapid washout. Heterogeneity in ultrasound systems, operator experience, and patient populations further underscores the need for standardized protocols. **Conclusions**: Overall, these data suggest that CEUS, particularly when combined with additional sonographic or cytological tools, significantly enhances diagnostic precision for peripheral pulmonary lesions.

## Simple Summary

Accurately identifying whether a lung lesion is benign (non-cancerous) or malignant (cancerous) is essential for choosing the best treatment. Contrast-enhanced ultrasound (CEUS) allows clinicians to observe how quickly blood enters and leaves the lesion, which can provide clues about its nature. Findings suggest that cancerous lesions typically receive blood later and lose it faster than benign ones. Some studies also showed that using this method during tissue sampling (biopsy) significantly improves the accuracy and safety of the procedure. While no single measurement was perfect, combining several blood flow characteristics gave highly reliable results. These insights may help clinicians make more confident diagnoses, reduce the need for repeat procedures, and improve patient outcomes. Overall, this method can enhance how peripheral lung lesions are assessed and managed, especially in patients who may not be suitable for more invasive diagnostic approaches.

## 1. Introduction

Peripheral pulmonary lesions represent a diagnostic challenge in pulmonary medicine. The prevalence and diagnostic challenges of peripheral pulmonary lesions vary globally. In the United States, administrative data show that pulmonary nodules affect approximately 0.34% to 0.70% of commercially insured individuals, with significantly higher rates—up to 5.97%—observed in the Medicare population, particularly among those over 45 years [1]. In China, a large-scale study involving 930 patients demonstrated that ultrasound-guided biopsy achieved a diagnostic accuracy of 90.3%, highlighting the growing clinical integration of ultrasound-based diagnostics in routine pulmonary care [2]. Low-dose computed tomography (CT) remains the most widely used initial imaging modality due to its high-resolution capabilities [3,4]. However, it involves radiation exposure and may be less feasible for certain patient groups. Positron emission tomography (PET), often combined with CT, provides functional imaging to assess metabolic activity and is essential for cancer staging. Its high cost and limited availability, however, can restrict access [5]. Percutaneous biopsy, guided by CT or ultrasound, is a minimally invasive method with high diagnostic yield. While CT-guided biopsy is common, it carries a higher risk of complications such as pneumothorax. Ultrasound-guided biopsy, particularly for lesions adjacent to the pleura, offers a safer alternative without radiation exposure and can be performed at the bedside [6]. This method is increasingly favored for its diagnostic precision and practicality.

Among the tools employed to characterize and diagnose these lesions [7], ultrasonography has traditionally played a supportive role, especially for those lesions abutting the pleura, given the inherent limitations of ultrasonography in deeper lung fields [8,9]. However, the advent of contrast-enhanced ultrasound (CEUS) has broadened the capability of ultrasound to distinguish between different vascular patterns, thus providing additional diagnostic insights [10,11]. Over the last decade, several studies have examined whether CEUS improves diagnostic accuracy when differentiating benign from malignant peripheral pulmonary lesions and whether it can enhance biopsy yield when combined with rapid on-site evaluation (ROSE) [8,9,12,13,14,15].

Quantitative CEUS is an advanced imaging technique that measures how injected microbubble contrast agents perfuse a target lesion or organ in real time [16,17,18]. By analyzing parameters such as arrival time, time to peak, wash-in/washout rates, and enhancement patterns, it provides objective, numerical data on vascularization [15,16]. This allows more precise lesion characterization, improves diagnostic accuracy, and can guide treatment decisions in various clinical settings [19,20].

This meta-analysis aims to bring together the findings from multiple studies investigating the use of quantitative CEUS in peripheral pulmonary lesions. We aimed to summarize the diagnostic accuracy, utility, and potential pitfalls of CEUS, focusing specifically on key parameters such as contrast arrival time (AT), washout time (WOT), perfusion pattern, and the role of combining CEUS with other modalities such as real-time on-site cytopathological assessment (e.g., ROSE). As a diagnostic systematic review, it was not registered in PROSPERO. We followed PRISMA reporting guidelines [21].

## 2. Methods

### 2.1. Eligibility Criteria

Included studies examined human subjects with peripheral pulmonary lesions undergoing quantitative CEUS analysis with or without biopsy correlation. Only full-text, peer-reviewed articles in English were considered.

### 2.2. Information Sources

PubMed/NIH, Embase, and the Cochrane Library were searched until December 2024.

### 2.3. Search Strategy

The search terms were combinations of the relevant medical subject heading (MeSH) terms, key words, and word variants for “lung”, “neoplasm”, and “contrast-enhanced ultrasound”. Each study’s title and abstract were examined first, and then the complete text was read to further filter the publications. Furthermore, a manual screening of each article’s references was conducted to find other potentially relevant studies.

### 2.4. Selection Process

Two independent reviewers screened titles and abstracts, followed by full-text analysis. Reference lists were manually searched.

### 2.5. Data Collection Process

Data were extracted independently by two authors using a standardized form, including study design, CEUS parameters, and diagnostic outcomes.

### 2.6. Data Items

From each study, the following variables were extracted when reported:

○ Study design (retrospective, prospective, or ambispective);
○ Number of patients and number of lesions;
○ Key CEUS parameters analyzed (e.g., arrival time (AT), time difference of arrival (TDOA), washout time (WOT), enhancement pattern, perfusion homogeneity);
○ Diagnostic criteria for malignancy (e.g., AT ≥ 10 s, lesion-lung AT difference ≥ 2.5 s, washout pattern, logistic regression models incorporating multiple features);
○ Sensitivity, specificity, and diagnostic accuracy;
○ Additional outcomes, such as biopsy success rate and procedure complications.

### 2.7. Study Risk of Bias Assessment

To assess the methodological quality of the included studies, the Quality Assessment of Diagnostic Accuracy Studies (QUADAS) tool was used by two researchers independently, which constituted 14 questions [22]. For each item, the study was rated as “yes” (high quality) if reported; “no” (low quality) if not reported; or “unclear” if no adequate information was provided. Disagreements were also resolved by a third researcher.

### 2.8. Effect Measures

Due to heterogeneity, no pooled estimates (e.g., ORs or summary ROC) were calculated. Instead, descriptive synthesis and individual AUC values were presented.

### 2.9. Synthesis Methods

A qualitative summary and structured tables are provided (Table 1, Table 2 and Table 3). No meta-analysis was performed due to non-uniform outcomes.

### 2.10. Synthesis Methods, Reporting Bias Assessment, and Certainty Assessment

Formal, quantitative meta-analytic pooling of effect sizes (e.g., via summary sensitivity, specificity, or likelihood ratios) requires substantial uniformity in outcome measurements, but many of the included studies instead provide qualitative or semi-quantitative results. Consequently, this systematic review largely employs a descriptive synthesis approach.

## 3. Results

### 3.1. Study Identification

**Figure 1:**
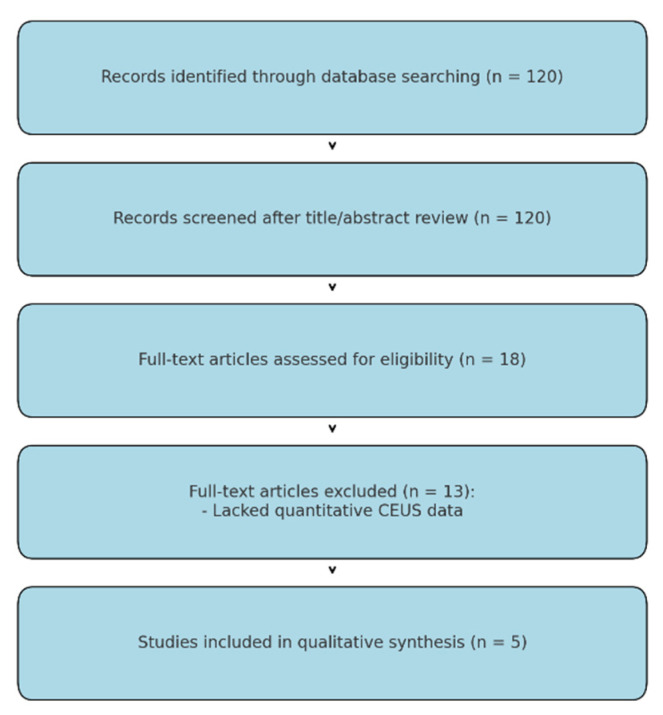
Study flow diagram.

A total of 120 relevant articles were detected in the initial search stage; many of these studies were excluded due to titles and abstracts. Only 18 studies were selected for full text review [12,13,14,28,29,30,31]. Further analysis excluded 13 studies lacking data on quantitative CEUS. Finally, 5 articles satisfying the inclusion criteria were included and analyzed [23,24,25,26,27] (Figure 1).

### 3.2. Quality Assessment

**Figure 2:**
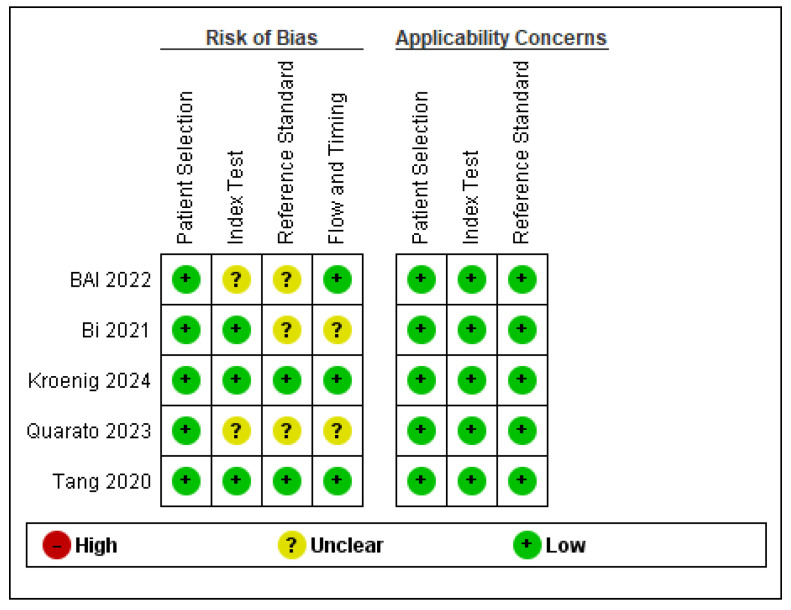
Risk of bias and applicability concerns summary: review authors’ judgements about each domain for each included study [23,24,25,26,27].

The methodological quality assessment of the included studies, summarized in Figure 2, demonstrates an overall low risk of bias and high applicability to the research question. All five studies exhibited low concerns across all domains related to applicability, including patient selection, index test, and reference standard. Regarding risk of bias, minor concerns were identified in a few studies: Bi (2021) [24] and Quarato (2023) [25] showed unclear risk in the “Index Test” domain, while Bai (2022) [23] presented some uncertainty in the “Reference Standard” and “Flow and Timing” categories. No study was rated as high risk in any domain, supporting the robustness and reliability of the evidence included in this systematic review.

### 3.3. Study Characteristics

In the study of Bai et al., 80 patients with peripheral pulmonary focal lesions were divided into two groups: a conventional ultrasound group versus a CEUS group [23] (Table 1). Both groups underwent diagnostic procedures guided by ROSE (rapid on-site evaluation) to improve biopsy yield [23]. The success rate of biopsy in the CEUS group reached 97.62%, significantly exceeding that of the conventional ultrasound group (84%) [23]. Additionally, complications such as pneumothorax or bleeding were assessed. While the difference in the incidence of complications between the two groups did not reach statistical significance (0% in the CEUS group vs. 5.26% in the conventional group), it nonetheless suggested that CEUS might be safer or at least non-inferior from a safety standpoint [23]. Moreover, within the CEUS group, the study analyzed several important time parameters: lesion enhancement, arrival time (AT) to the lesion, lung arrival time (L-AT), and difference in AT (∆AT) = L-AT − AT. A significant result was that the difference (∆AT) helped discriminate between benign and malignant lesions [23]. Specifically, a threshold of 2.05 s for ∆AT was identified as optimal in differentiating the two categories [23]. These findings underscore that time-based indices in CEUS, in combination with real-time needle guidance (and immediate cytological feedback), can dramatically improve diagnostic yield.

Bi et al. performed an ambispective cohort study composed of a development cohort (DC) of 592 patients and a validation cohort (VC) of 220 patients [24]. Eighteen parameters from both B-mode ultrasound and CEUS were evaluated [24]. Using binary logistic regression, the authors constructed a predictive model that integrated six parameters: angle between lesion border and thoracic wall, basic intensity, lung-lesion arrival time difference, ratio of arrival time difference, vascular sign, non-enhancing region type.

This composite model was then validated both internally (in the DC) and externally (in the VC). Its performance was outstanding, with a C-statistic (i.e., AUC) of 0.974 internally and 0.980 externally [24]. This far surpassed the C-statistics for the simpler, widely used criteria of lesion-lung AT difference ≥ 2.5 s (C-statistic: 0.842 in the DC and 0.777 in the VC) and AT ≥ 10 s (C-statistic: 0.688 in the DC and 0.641 in the VC).

These differences were statistically significant (*p* < 0.001) [24]. In terms of raw diagnostic performance, the multi-parameter model exhibited a sensitivity of 94.82% (DC) and 92.86% (VC) and a specificity of 92.42% (DC) and 92.59% (VC) [24]. Both metrics were superior to simpler single-parameter approaches [24].

This study thus demonstrated that combining B-mode sonographic features (like angle between lesion and thoracic wall) with CEUS parameters (like arrival time, washout patterns, or vascular signs) can achieve high diagnostic accuracy [24].

Quarato and colleagues sought to address the debated value of contrast enhancement arrival time in differentiating benign from malignant peripheral pulmonary lesions [25]. A total of 317 patients (215 men, 102 women; mean age 52) were included, all of whom underwent pulmonary CEUS with sulfur hexafluoride microbubbles [25].

Arrival time (AT) alone was not shown to differ substantially between benign and malignant lesions. In fact, setting a cutoff of <10 s as indicative of benignity had low diagnostic accuracy (47.6%) and very poor sensitivity (5.3%) [25].

The authors also looked at washout time (WOT) with a cutoff of >300 s. This, similarly, yielded a low accuracy (53.6%) and 16.5% sensitivity. These results were consistent across sub-analyses by lesion size (smaller vs. larger than 3 cm) [25].

Interestingly, among malignant lesions, squamous cell carcinoma had a more delayed AT compared to other histopathologic subtypes. This difference, however, only reached statistical significance when compared to undifferentiated lung carcinomas [25].

The study’s overall conclusion was that arrival-time-based criteria alone offer limited discriminatory power [25]. This somewhat contradicts or tempers the more optimistic thresholds reported by other authors. Quarato et al. emphasize that, while CEUS can provide real-time vascular insight into peripheral lesions, arrival time alone may not be adequate in capturing the complexities that differentiate pneumonia, inflammation, or necrosis from malignant infiltration [25].

Tang and colleagues retrospectively examined 96 patients with PPLs, comparing conventional time-intensity curve (TIC) indices to a new index called the time difference of arrival (TDOA) [26]. Notably, TDOA is conceptually similar to ∆AT in that it compares the timing of contrast arrival between the lesion and a reference structure (often normal lung or pleura) [26]. Malignant lesions demonstrated a markedly higher TDOA relative to benign lesions (*p* < 0.001) [26]. TDOA had an area under the ROC curve of 0.894, indicating robust accuracy for differentiating malignant from benign inflammatory lesions [26]. Conventional CEUS indices (e.g., peak enhancement, washout slope, etc.) did not improve upon TDOA’s accuracy once TDOA was used as the primary parameter [26].

Thus, Tang et al.’s data support the notion that a time-based index—when carefully defined—has real diagnostic utility [26]. They theorized that malignant lesions have a more pronounced and delayed vascular supply (often from bronchial arteries with higher resistance and lower flow) compared to inflammatory processes that may recruit faster, more abundant infiltration of microbubbles, or a different vascular bed dynamic [26].

Kroenig and colleagues focused on 54 patients with histologically proven peripheral pulmonary metastases [27]. The CEUS parameters of interest included: time to enhancement (TE), categorized as early pulmonary-arterial (PA) or delayed bronchial-arterial (BA) patterns; extent of enhancement (EE), either marked or reduced; homogeneity of enhancement (HE); decrease of enhancement (DE); and rapid washout (<120 s) or late washout (≥120 s). Interestingly, most lesions (92.6%) showed a BA (bronchial artery) enhancement pattern, aligning with the concept that metastatic lesions often rely on systemic (bronchial) rather than pulmonary circulation [27]. Moreover, 98.1% showed a rapid washout pattern (<120 s) [27]. Immunohistochemical staining with CD34 confirmed that the vascular supply was chaotic and predominantly from the bronchial circulation in nearly all cases [27]. Even the four lesions that initially seemed to enhance via a pulmonary-arterial pattern were ultimately shown to have an aberrant or mixed vascular supply [27]. Therefore, CEUS might detect chaotic or aberrant vascular patterns typical of neoplasia. Pulmonary metastases, like primary malignancies, rarely rely on normal alveolar or pulmonary arterial flow alone. Thus, CEUS findings of extensive or early enhancement with subsequent rapid washout are consistent with the presence of neoplastic tissue, something that may help exclude purely inflammatory or infectious processes [27].

## 4. Synthesis of Findings

The included studies highlight that CEUS is a valuable tool in the evaluation of peripheral pulmonary lesions but that its utility is not uniformly summarized by a single parameter (Table 2). Some studies have found that simple thresholds, such as an AT ≥ 10 s or a lesion-lung AT difference ≥ 2.5 s, can be fairly specific yet not always sufficiently sensitive [24]. Others have shown that more sophisticated or nuanced measurements—like ∆AT (the difference in arrival times between the lesion and lung or pleura), TDOA, or logistic regression models integrating multiple parameters—significantly enhance diagnostic performance [23,26].

Single-parameter thresholds, such as AT ≥ 10 s, provide moderate specificity but may miss a significant proportion of malignant lesions, leading to lower sensitivity and overall diagnostic accuracy [25]. Multi-parameter models, as demonstrated by Bi et al., achieve near-excellent discrimination between malignant and benign lesions, with C-statistics approaching 0.98 [24]. This suggests that no single CEUS parameter can fully characterize the complexity of a lesion’s vascularity, but multiple combined features—both B-mode and contrast-enhanced—offer a robust tool.

The difference in arrival times (lesion vs. adjacent lung tissue) has emerged in multiple studies as a strong discriminator of malignancy [23,24,25,26,27]. Malignant lesions typically show a delayed but more intense supply from higher-pressure systemic circulation (bronchial arteries), whereas benign inflammatory lesions can be perfused more quickly via alveolar or partially compromised alveolar circulations. Different numeric thresholds have been proposed for TDOA or ∆AT (ranging from around 2 s to as high as 2.5 s) [23,26]. These small differences in cut-off might be explained by different ultrasound system settings, patient selection, or definitions for reference arrival time. The concept of washout time in CEUS is well documented in liver and other organ lesions, where a prolonged washout is often associated with malignancy. However, in the lung, the data are mixed. Quarato et al. reported that WOT (> 300 s) was not a particularly good discriminator for benign vs. malignant lesions (53.6% diagnostic accuracy, 16.5% sensitivity) [25]. On the other hand, in metastatic lesions, as Kroenig et al. noted, a rapid washout was present in almost all cases (98.1%) [27]. These divergences may be related to histological differences or possibly to how malignant lesions in the lung are vascularized. Primary malignant lung tumors, inflammatory lesions, and metastases from different primary sites could all behave differently with respect to contrast washout.

Moreover, CEUS can significantly improve the success rate of biopsy (97.62% vs. 84% with conventional ultrasound) [23]. This improvement may stem from CEUS’s ability to better delineate viable and vascularized parts of the lesion, reducing the likelihood of sampling necrotic or non-diagnostic areas. The combination of CEUS with ROSE offers immediate cytopathological feedback, further enhancing the yield and potentially reducing the risk of complications if fewer passes are needed to obtain diagnostic tissue [23]. Concerning histopathological correlations, Quarato et al. noted that squamous cell carcinoma had a more delayed arrival time compared to other histological subtypes [25]. Meanwhile, Kroenig et al. used immunohistochemical CD34 staining to corroborate that metastatic lesions predominantly displayed a bronchial artery blood supply (chaotic or aberrant vascular patterns) [27]. These nuances underscore that CEUS patterns may even be predictive of specific pathological subtypes, although larger confirmatory studies are needed.

## 5. Discussion

Across the studies, one consistent theme is that malignant lesions typically exhibit a more pronounced bronchial arterial supply, leading to a delayed but robust enhancement and a faster washout phase on CEUS. By contrast, benign lesions—especially inflammatory or infectious lesions—may show earlier arrival of contrast (through alveolar vessels or less chaotic neo-vasculature) or different washout characteristics. However, as Quarato et al. argued, these findings are not universal, and arrival time alone is not foolproof for discrimination [25].

CEUS can help to identify the most viable region of a lesion for biopsy, thereby potentially enhancing diagnostic yield, particularly when combined with on-site pathological evaluation. The improved success rates noted by Bai et al. (97.62% vs. 84%) suggest that fewer repeat procedures may be necessary, thereby reducing overall patient risk and associated costs [23]. In patients who may be poor candidates for more invasive diagnostic approaches (e.g., those with advanced emphysema or other comorbidities), CEUS-guided biopsy provides a less invasive alternative that can still offer high diagnostic accuracy.

The multi-parameter logistic regression model [24] underscores a crucial point: no single feature of CEUS, or even B-mode ultrasound, can always reliably distinguish benign from malignant. However, a composite approach is extremely powerful. In a previous systematic review and meta-analysis, our group demonstrated that the simultaneous evaluation of multiple CEUS features allows one to reach an excellent diagnostic accuracy [32]. Non-homogeneous CE with early washout were the most indicative features of malignancy of a lung lesion [32].

For patients who have borderline contraindications to biopsy or for those in whom immediate histological confirmation may be difficult, an accurate risk stratification tool has obvious clinical value. One might, for example, decide to proceed with a biopsy if the model indicates high risk of malignancy or consider a trial of antibiotics to consolidate suspicion of infection if the model supports a benign etiology [33].

Different manufacturers’ ultrasound platforms have varying sensitivity and algorithms for quantifying arrival time or washout times. Additionally, though all the cited studies utilized second-generation ultrasound contrast agents (e.g., sulfur hexafluoride microbubbles stabilized by phospholipid shells), small differences in injection techniques, dosage, and patient circulation dynamics could influence measured times [23,24,25,26,27].

Malignancies can differ widely by histological subtype (e.g., adenocarcinoma vs. squamous cell carcinoma, primary lung cancer vs. metastatic lesion) [34]. Studies focusing on a single lesion type (like Kroenig et al. with metastases [27]) may find consistent vascular patterns that are distinct from those found in a more heterogeneous population. Therefore, the discriminatory power of a given threshold might vary widely depending on the distribution of pathologies in a study.

Therefore, CEUS can provide real-time visualization of microvascular perfusion using intravenous microbubbles, allowing for the dynamic assessment of blood flow patterns within pulmonary lesions. Unlike CT or MRI, which may require contrast agents with nephrotoxic risk or expose patients to ionizing radiation, CEUS is radiation-free and can be safely repeated. Furthermore, CEUS can be performed at the bedside and offers high temporal resolution, making it particularly useful for evaluating peripheral lung lesions that are in contact with the pleura. These features contribute to its superior performance in distinguishing between benign and malignant lesions, especially when combined with real-time guidance for biopsy.

## 6. Limitations of the Current Evidence Base

Some studies did not specifically mention whether the sonographers were blinded to prior imaging (CT or PET-CT) or to the clinical suspicion of malignancy. A large proportion of studies used retrospective cohorts, which can introduce selection bias. While Bi et al. [24] used a prospective validation cohort, the majority of patients in that study were still from a retrospective “development” set. Sub-analyses of specific histological types (e.g., squamous cell carcinoma or metastatic lesions from certain primaries) often involved relatively small numbers. Drawing robust statistical conclusions from these small groups can be unreliable, and further validation in larger prospective cohorts is needed. Studies used different endpoints such as sensitivity, specificity, area under the ROC curve, or overall accuracy. They also varied in how they defined benign lesions (clinical-radiological resolution vs. histopathological confirmation) and malignant lesions. This heterogeneity complicates direct comparisons and meta-analytic pooling of results.

## 7. Future Directions

In order to validate thresholds like TDOA or multi-parameter logistic models, well-designed multicenter trials with standardized CEUS protocols are necessary. A concerted effort to unify definitions of AT, TDOA, WOT, and so forth would greatly clarify their real-world reliability. Given the success of logistic regression in combining multiple sonographic parameters, it is plausible that more sophisticated AI approaches (e.g., machine learning, neural networks) could further improve accuracy. Automated detection and quantification of lesion enhancement characteristics might eventually reduce operator variability and speed up the diagnostic process. While combining CEUS with ROSE or B-mode ultrasound has proven beneficial, there is also potential for synergy with CT or PET. For instance, comparing CEUS TDOA data with PET-CT metabolic uptake might offer a powerful combined biomarker of malignancy. Beyond diagnosis, it remains to be seen whether CEUS can play a role in monitoring tumor response to therapies such as immunotherapy or targeted agents. Changes in lesion vascularization patterns, as detected by CEUS, might precede radiological size changes.

## 8. Conclusions

This meta-analysis highlights the growing body of evidence supporting quantitative CEUS as a valuable diagnostic modality for peripheral pulmonary lesions. While conventional ultrasound has well-recognized limitations in pulmonary imaging, CEUS offers critical additional information about lesion vascularity, potentially distinguishing benign inflammatory or infectious processes from primary lung cancers and metastatic disease. In conclusion, the body of evidence presented in these studies collectively demonstrates that CEUS is of high value in distinguishing benign from malignant peripheral pulmonary lesions and in guiding percutaneous biopsy. CEUS can reduce diagnostic uncertainty, minimize complications, and potentially allow clinicians to make timely and accurate treatment decisions for patients with peripheral pulmonary lesions.

Bullet Points:

- When used in tandem with ROSE, CEUS-guided biopsy can achieve success rates approaching 98%, significantly higher than conventional ultrasound-guided approaches.
- Time difference of arrival (∆AT/TDOA) consistently emerges as a useful discriminator between benign and malignant processes, with different proposed numeric thresholds (2.05–2.5 s) providing strong sensitivity and specificity.
- Multi-parameter models that integrate B-mode features (lesion shape, angle with chest wall, vascular sign) and CEUS features (arrival time difference, basic intensity, presence of non-enhancing regions) can achieve near-perfect discrimination, with AUC values > 0.95 in both developmental and external validation cohorts.

## Figures and Tables

**Table 1:** Summary of findings.

Author(s)	Population Size	Study Type	Key Diagnostic Tools	Diagnostic Accuracy/AUC	Main Conclusion
Bai et al. [23]	80	Prospective comparative	CEUS + ROSE	Biopsy success: 97.62% (CEUS) vs. 84% (conventional)	CEUS improves biopsy yield; ΔAT helps discriminate lesions
Bi et al. [24]	812	Ambispective cohort (development + validation)	B-mode + CEUS (multi-parameter model)	AUC: 0.974 (dev), 0.980 (val); sens: ~93–95%, spec: ~92%	Multi-parametric model significantly outperforms single thresholds
Quarato et al. [25]	317	Retrospective	CEUS (AT, WOT)	AT <10 s: 47.6% accuracy, WOT >300 s: 53.6% accuracy	AT and WOT alone are unreliable discriminators
Tang et al. [26]	96	Retrospective	CEUS (TDOA)	AUC: 0.894 for TDOA	TDOA is a robust parameter to distinguish malignancy
Kroenig et al. [27]	54	Observational retrospective	CEUS (TE, EE, HE, DE) + CD34 staining	92.6% BA pattern, 98.1% rapid washout (<120 s)	Metastases show BA supply and rapid washout, supporting CEUS utility

**Table 2:** Summary table of selected studies. Abbreviations: AT, arrival time; L-AT, lung arrival time; ΔAT; difference between L-AT and AT; TDOA, time difference of arrival; WOT, washout time; DC, development cohort; VC, validation cohort.

Reference	Study Design	Population and Sample	Main Ceus Parameters	Key Results
Bai Z et al. (2022) [23]	Prospective study	- 80 patients with peripheral pulmonary lesions - Divided into a conventional ultrasound group vs. a CEUS group, both using real-time ROSE	- Lesion enhancement - Arrival time (AT) - Lung arrival time (L-AT) - ΔAT (difference in arrival time)	- Biopsy success rate: 97.62% (CEUS) vs. 84% (conventional US) - Optimal ΔAT threshold = 2.05 s to distinguish benign vs. malignant - No significant complications in the CEUS group vs. 5.26% in the conventional group.
Bi K et al. (2021) [24]	Ambispective (retro-/prospective)	- Development: 592 patients (DC cohort, 2017–2018) - Validation: 220 patients (VC cohort, 2019) - 18 parameters from B-mode US and CEUS were collected	- Lesion-lung arrival time difference - AT ≥ 10 s (historical criterion) - Multivariate model (6 parameters)	- Final model with 6 parameters (B-mode + CEUS) with C-statistic: 0.974 (DC) and 0.980 (VC) - Superior to AT ≥ 10 s or difference ≥ 2.5 s - Sensitivity ~93–95% and specificity ~92%.
Quarato Cmi et al. (2023) [25]	Retrospective study	- 317 patients (215 men, 102 women; mean age 52) - Peripheral pulmonary lesions (benign/malignant)	- Arrival time (AT) - Enhancement pattern - Washout time (WOT)	- AT < 10 s vs. ≥ 10 s did not effectively differentiate benign vs. malignant (low sensitivity, 5.3%) - WOT > 300 s was not discriminative (accuracy ~53.6%) - Squamous cell carcinoma displayed a later AT than other subtypes, but significant difference only vs. undifferentiated carcinoma.
Tang M et al. (2020) [26]	Retrospective study	- 96 patients with peripheral pulmonary lesions undergoing biopsy - Comparison between conventional CEUS parameters and a new TDOA index	- TDOA (time difference of arrival) - Time-intensity curve (TIC) parameters	- TDOA was significantly higher in malignant lesions (*p* < 0.001) - AUC for TDOA = 0.894, outperforming conventional CEUS parameters
Kroenig J et al. (2024) [27]	Retrospective study	- 54 patients with histologically proven peripheral pulmonary metastases - Included cases with immunohistochemical correlation (CD34)	- Enhancement time (TE): pulmonary-arterial (PA) or bronchial-arterial (BA) pattern - Extent and homogeneity of enhancement - Washout (<120 s or ≥120 s)	- 92.6% had a BA pattern, 98.1% exhibited a rapid washout - A “chaotic” vascular pattern correlated with tumor neo-angiogenesis (CD34) - Only 4 lesions (7.4%) showed a PA pattern.

**Table 3:** Description of quantitative CEUS parameters evaluated across different studies.

**Parameter**	**Description**
AT (Arrival Time)	The time at which the contrast agent first appears in the target lesion.
L-AT (Lung Arrival Time)	The time at which the contrast agent first appears in the adjacent lung tissue.
ΔAT (Difference Between L-AT AND AT)	This parameter measures the discrepancy in arrival times between healthy lung and lesion; calculated as ΔAT = L-AT − AT. It helps assess differences in blood flow between normal lung and the lesion.
TDOA (Time Difference OF ARRIVAL)	Conceptually similar to ΔAT, it compares the time of contrast arrival in the lesion versus a chosen reference structure. TDOA is often used in evaluating perfusion delays indicative of malignancy.
WOT (Washout Time)	The time required for the contrast signal to disappear from the lesion (the washout phase). This can provide information on lesion vascularity and potential malignancy markers.

Registration and protocol: diagnostic systematic review are not registered in PROSPERO.

## Data Availability

Data are available on request from the corresponding author.
